# Response to: Correspondence on 'Novel imaging biomarkers predict outcomes in stage III unresectable non-small cell lung cancer treated with chemoradiation and durvalumab' by Zheng *et al*

**DOI:** 10.1136/jitc-2022-005086

**Published:** 2022-05-31

**Authors:** Vidya Sankar Viswanathan, Mohammadhadi Khorrami, Khalid Jazieh, Pingfu Fu, Nathan Pennell, Anant Madabhushi

**Affiliations:** 1 Department of Biomedical Engineering, Case Western Reserve University, Cleveland, Ohio, USA; 2 Biomedical Engineering, Case Western Reserve Univ, Cleveland, Ohio, USA; 3 Department of Internal Medicine, Cleveland Clinic, Cleveland, Ohio, USA; 4 Population and Quantitattive Health Sciences, Case Western Reserve University, Cleveland, Ohio, USA; 5 Department of Hematology and Medical Oncology, Taussig Cancer Institute, Cleveland Clinic, Cleveland, Ohio, USA

**Keywords:** Immunotherapy, Tumor Biomarkers

Dear Editor,

Dr Zheng *et al* have mentioned several concerns with the methods and approaches in our study.^1^ First, they have suggested the use of sophisticated and rigorous dimensionality reduction methods for validation. We would like to point out that least absolute shrinkage and selection operator (LASSO), the method used in our paper, is a standard approach for identifying features, which allows strongly correlated features to be selected. Our current approach was influenced by past successes in implementation and validation of prognostic, predictive imaging algorithms using LASSO. While we acknowledge that several other approaches could have been employed and evaluated for identifying the most discriminating features, the optimal model choice for feature selection was beyond the scope of our current study.^2–13^

In the letter, Zheng *et al* have also suggested that the survival differences be evaluated using landmark test instead of log-rank test. While landmark-based predictions are useful for patient outcome at discrete time points, our study included predetermined groups. We tested the ability of the biomarker to stratify high-risk subjects and low-risk subjects over the entire duration of follow-up, as opposed to risk stratification at a fixed time point.^14^

Zheng *et al* also state that we did not explicitly report the features that comprised our radiomics signature. However, we draw their attention to the Discussion section in the paper, where we mentioned that Laws and Haralick features (intratumoral region) and Gabor and Laws features (peritumoral region) were the most prognostic features.

Dr Zheng *et al* suggest employing other indicators of predictive performance, in addition to C-index. We used C-index as it is a commonly used and well-accepted metric for evaluating overall model performance, and other discrimination metrics such as net reclassification index are complementary to C-index.

We agree with Dr Zheng *et al* that scanner parameters can influence the extracted radiomics features. We acknowledge that this was indeed a limitation of the study, though we have evaluated the impact of acquisition-related parameters on radiomics in a number of publications^15 16^ we cited in our paper.^15 17^

Another issue pointed out has been regarding the potential selection bias in cohort D3 in terms of their age, sex, race, and smoking. We acknowledge that the demographics of D3 differed from the other cohorts since these patients belonged to the veteran population and were from the VA Healthcare system. Lung cancer is the leading cause of death among the veterans; hence, in our study, we sought to evaluate the utility of radiomics in this population.

We agree with the point that treating PD-L1 scores as a continuous measure could be more efficient under the assumption that the effect of PD-L1 scores is the same across the whole spectrum of PD-L1. However, that assumption does not likely hold and, as a result, various cut-off points of PD-L1 scores have been used in published studies. The 50% tumor proportion score (TPS) cut-off defines a widely accepted biomarker subgroup with a better prognosis,^18 19^ while lower levels of PDL1 have not been shown to have clearly different outcomes based on levels. Hence, from a clinical utility perspective, we employed the 50% TPS cut-off for our study.

Zheng *et al* also raised a concern that the number of non-smokers was far less than smokers, and that there was no significant difference in smoking status between high-risk and low-risk patients in our study; hence, this factor could be excluded from the nomogram. While our experiment did not find any significant association of smoking with risk groups, previous studies have demonstrated smoking to be an independent prognostic factor in lung cancer.^20 21^ Hence, we included smoking in our clinical and combined predictive model.

Finally, the authors have pointed out that the biological rationale of radiomic biomarkers have not yet been clarified and suggested that a possible mechanism to suboptimal response with durvalumab might be immune checkpoint inhibitors (ICI) resistance. We would like to underscore the strength of radiomics in analyzing the tumor in its entirety, considering the spatial and temporal heterogeneity over tissue-based biomarkers which examine only a small section of the sample. In addition, our group has extensively analyzed the association of tumor biology and histopathology-based features with radiomic features. In our previous study,^12^ we had access to the biopsied tissue from patients with non-small cell lung cancer treated with ICI therapy. To investigate associations between the radiomic features and density of tumor-infiltrating lymphocytes (TILs), we employed automated nuclei detection followed by density estimation of TILs in diagnostic digitized H&E images. Our study revealed that TIL density was significantly (ρ=−0.5, p<0.05) correlated with peritumoral Gabor features from the first annular ring outside the nodule.

While PD-L1 is the gold standard biomarker to select patients for treatment with ICI, its performance to identify patients receiving maximum benefit is poor. Our study evaluated the radiomic nomogram of patients by decision curve analysis and calculated the net benefit of ICI. The strength of our study lies in the ability of the radiomic signature to predict patients with a higher overall benefit from ICI than the clinical–pathological measurements which holds true across the spectrum of PD-L1 expression. Despite the clinical benefit and utility, much work needs to be done for clinical deployment of these tools. The radiomic tools must be evaluated on a larger, multi-institutional dataset, which accounts for population-based differences to ensure that these tools do not inadvertently introduce bias. Prospective trials, either non-interventional or interventional, is the gold standard for validation studies and would demonstrate the strongest evidence for radiomics as predictive biomarker in clinical practice.
